# Impact of the risk-adapted Nordic anal cancer group consensus guidelines on the contouring of the elective clinical target volume in anal cancer

**DOI:** 10.2340/1651-226X.2025.42723

**Published:** 2025-05-26

**Authors:** Marcus Johnsson, Sara Alkner, Anders Johnsson, Martin P. Nilsson

**Affiliations:** a Division of Oncology and Pathology, Department of Clinical Sciences, Lund University, Lund, Sweden; b Department of Hematology, Oncology and Radiation Physics, Skåne University Hospital, Lund, Sweden

**Keywords:** Anal carcinoma, radiotherapy, delineation, contouring guidelines, lymph node metastasis

## Abstract

**Background and purpose:**

The recently published Nordic anal cancer group (NOAC) contouring guidelines aim for improved oncological outcomes and reduced toxicity in anal cancer patients treated with radiotherapy. The present work describes how the elective clinical target volume (CTVe) would change when applying the NOAC guidelines instead of the previous Australasian standard. According to the Australasian guidelines, the cranial border of the CTVe is at the common iliac bifurcation for all patients, and the external iliac region as well as the ischiorectal fossa are always included.

**Materials and methods:**

Retrospectively, 166 anal cancer patients treated with curative radiotherapy according to Australasian guidelines between 2009 and 2017 were studied in a single-center analysis. Pretherapeutic scans, and clinical information were used to categorize patients according to the NOAC guidelines for a comparison with the Australasian guidelines.

**Results:**

Applying the risk-adapted alternative of the NOAC guidelines had the external iliac region omitted in 41.0% of the patients. The cranial border was lowered from the common iliac bifurcation in 27.7% and elevated in 12.7% of the patients. Elderly patients (≥70 years) more often had the external iliac region omitted than younger patients (60.9% vs. 33.3%; *p* = 0.001). The entire ischiorectal fossa was included in 23.7% of the patients due to tumor extension beyond the levator ani muscles or external sphincter.

**Interpretation:**

Contouring according to the NOAC risk-adapted guidelines changed, and mainly reduced, the CTVe in about half of all patients. Prospective follow-up is needed to determine if this is clinically beneficial.

## Introduction

Squamous cell carcinoma of the anal region (anal cancer) is a rare malignant disease with increasing incidence [1, 2]. Being organ preserving, chemoradiotherapy (CRT) is the recommended curative treatment for most patients for decades. Refinement of radiotherapy techniques and better diagnostic work-up have improved the prognosis, and today more than 80% of patients are cured [3–5]. However, due to the radiation dose to surrounding organs at risk (OAR), many patients will suffer from long-term side effects affecting their quality of life, for example, fecal incontinence, pain, and sexual dysfunction [6–8]. The risk of developing acute as well as long-term gastrointestinal toxicity has been reduced with intensity-modulated radiotherapy (IMRT) and seems to correlate with the bowel volume exposed to radiation [9–13]. A way of reducing radiation to OAR and improve treatment outcome could be to adjust the elective clinical target volume (CTVe) to the individual patient’s risk of recurrence. Even though such a treatment strategy is commonly applied in other squamous cell carcinomas, for example, cervical and oropharyngeal carcinoma, it has previously not been widely used in anal cancer [14–17].

In 2023, the Nordic anal cancer group (NOAC) published contouring guidelines which are the first to include a risk-adapted alternative for anal cancer patients (hereafter, the risk-adapted alternative is referred to as ‘the NOAC guidelines’) [18]. For patients without distant metastases, the NOAC guidelines suggest four different levels of the cranial border of CTVe: the ‘Very low’ border for T1-2N0 tumors not extending into the rectum; the ‘Low’ border for T1-2N0 tumors extending <1 cm into the rectum; the ‘High’ border for patients with lymph node (LN) metastasis in ≥3 pelvic or inguinal LN regions, and for patients with external or internal iliac LN metastasis in the upper half of those regions; and the ‘Intermediate’ border for all other patients. Furthermore, the NOAC guidelines recommend omission of the external iliac region for patients with tumors staged T1-2N0-1 with LN metastasis confined to the perirectal stations (along the superior rectal artery and the mesorectal and presacral regions). Finally, the NOAC guidelines suggest three different options for the delineation of the ischiorectal fossa (IRF), based on how the tumor is growing in relation to the levator ani muscles and the external sphincter. Table 1 shows how the NOAC guidelines differ from the Australasian, Radiation Therapy Oncology Group (RTOG) and UK contouring guidelines, respectively.

**Table 1:** Comparison between NOAC and other contouring guidelines regarding the CTVe.^1^

	NOAC	Australasian	RTOG	UK
**Cranial border**	4 risk-adapted levels based on T and N stage^2^	Bifurcation of the common iliac artery^3^	Bifurcation of the common iliac artery^3^	20 mm above the inferior aspect of sacroiliac joint^4^
**Inclusion of the external iliac region**	All except for T1-2N0-1a (perirectal)	Always	Always	Always
**Ischiorectal fossa (IRF)**	3 risk-adapted levels based on tumor growth	Entire IRF	A few mm beyond levator ani muscles	No extra margin beyond primary tumor CTV (CTVp)^5^

1 Elective clinical target volume.

2 See text under *Introduction.*

3 Corresponds to NOAC *Intermediate.*

4 Corresponds to NOAC *Low.*

5 Entire IRF is advised when the tumor grows > 5 mm beyond the levator ani muscles/external sphincter.

At our tertiary cancer center, 166 consecutive anal cancer patients were treated with curative intent ‘one size fits all’ radiotherapy according to RTOG/Australasian guidelines during the years 2009 through 2017. We have previously reported a favorable (86%) 5-year anal cancer specific survival, but a high rate (40%) of late grade ≥ 2 gastrointestinal toxicity in that cohort [9, 19]. The aim of the present study was to use the same cohort of patients and retrospectively simulate how the contouring of the CTVe would have changed if they instead had been treated according to the risk-adapted NOAC guidelines.

## Materials and methods

### Study population and data collection

**Figure 1:**
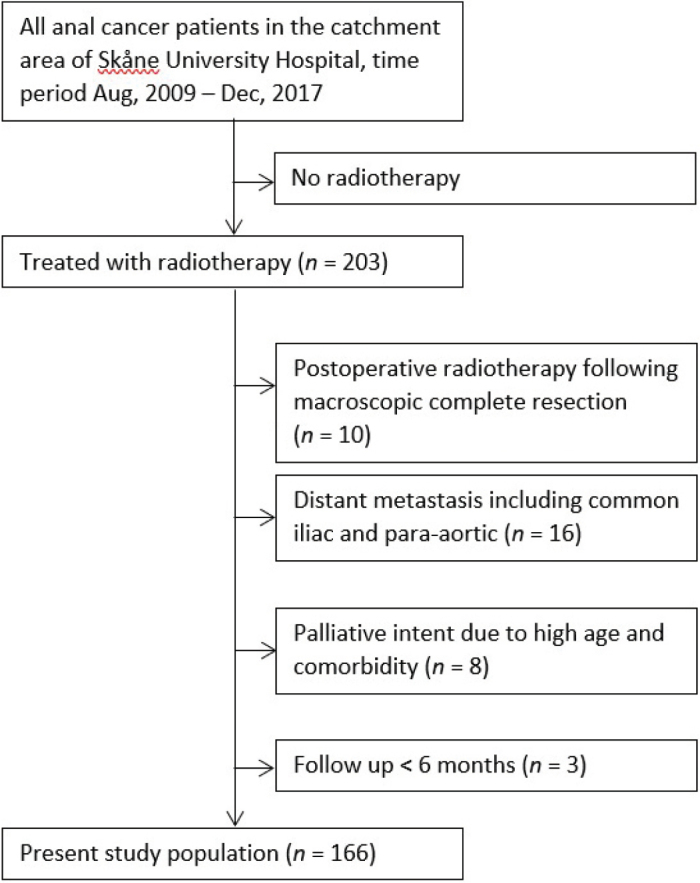
Flowchart of the study population.

The study population has been described in detail previously [19]. Briefly, all consecutive anal cancer patients treated with radiotherapy at the Skåne University Hospital during the period 2009–2017 were identified (*n* = 203). Following the exclusion of patients with palliative intent, distant metastasis (including common iliac and para-aortic LNs) at diagnosis, no macroscopic tumor left after primary surgery and <6 months of follow up, 166 patients remained (Figure 1). As part of a previous publication, data on patient and tumor characteristics were extracted from medical records. The presence of metastatic LNs was based on the judgement and radiological staging made by the treating physicians at the time of diagnosis. For the present study, diagnostic magnetic resonance imaging (MRIs) were reviewed to analyze the primary tumor, and radiotherapy planning computerized tomographs (CTs) were assessed to define the exact location of external iliac and internal iliac LN metastases. Furthermore, all patients were categorized into different subgroups according to the NOAC guidelines.

### MRI assessment

Diagnostic MRIs were accessible in 131 patients and were retrospectively reviewed by a clinical oncologist (MJ) to assess factors of importance for the CTVe according to the NOAC guidelines. All borderline cases were also evaluated by a radiation oncologist (MPN). Three categories were defined to describe how far cranially the primary tumor extended into the rectum:

1. No rectal involvement
2. Tumor extension <1 cm into the rectum
3. Tumor extension ≥1 cm into the rectum

**Figure 2:**
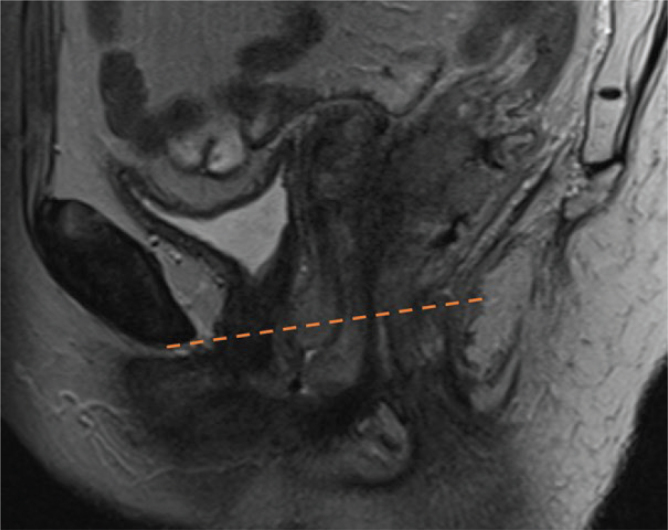
The anorectal junction was defined as the superior border of the puborectalis muscle. Tumor growth above the red line was considered within the rectum.

Patients with no accessible MRI (*n* = 35) were instead categorized using the clinical description at diagnosis combined with diagnostic CT and positron emission tomography (PET). Tumor extension into the rectum was defined as tumor growth above the superior border of the puborectalis muscle (Figure 2).

Three categories were also defined to describe the primary tumor’s growth in relation to the levator ani muscles and the external sphincter:

1. No extension into the levator ani muscles or the external sphincter
2. Tumor extension into, but not beyond, the levator ani muscles or the external sphincter
3. Tumor extension beyond the levator ani muscles or the external sphincter

Patients with no accessible MRI were not assessed regarding tumor growth in relation to IRF.

### External and internal iliac lymph node metastases

To decide on whether an iliac LN metastasis was situated in the upper or lower pelvic region, the radiotherapy planning CT was reviewed. The superior border of the pelvic region was defined as the bifurcation of the common iliac artery (first CT-slice with visible fat tissue between the internal and external iliac arteries) on the side of the most cranial iliac metastasis. The inferior border of the pelvic region was defined as the inguinal ligament (where the pelvic brim turns medially), in accordance with the NOAC guidelines. If the center of the LN metastasis was closer to the superior border of the pelvic region than the inferior, it was considered as an upper pelvic LN metastasis.

### Aims and statistical analysis

The primary aim of the study was to categorize the study population according to the following main features of the NOAC guidelines:

1. Level of the cranial border
2. Omission or inclusion of the external iliac region
3. Omission or inclusion of the entire IRF

A comparison was made with the 2012 Australasian guidelines, which often have been considered an international standard and were used at our institution until 2023 [16]. According to the Australasian guidelines, the cranial border should be at the bifurcation of the common iliac artery for all patients (corresponding to the ‘intermediate’ NOAC cranial border), and the external iliac region and the ischiorectal fossa should always be included (Table 1).

The study also aimed to evaluate whether applying the NOAC guidelines would impact target volumes in the following predefined subgroups: men versus women and older (≥70 years) versus younger patients.

Descriptive statistics expressed in percentages were used to present the differences between applying the NOAC guidelines and the Australasian guidelines. Statistical analyses on subgroups were done in SPSS version 29 using crosstabs, and statistical significance was assessed with the chi-squared test. *P*-values < 0.05 were considered significant.

### Sensitivity analysis

Decision on LN involvement was based on the clinical assessment that was made at the time of treatment, mainly incorporating findings on MRI and PET-CT, but without any strict and unequivocal criteria used for all patients. Therefore, a pre-planned sensitivity analysis was performed to estimate the robustness of the results. The baseline PET-CTs of the same cohort of patients have previously been assessed retrospectively, without taking the clinical situation into account, to map regional LN metastases using both Deauville score ≥ 3 and ≥ 4 as cut-offs for malignant nodes [20]. Deauville score is a scale from 1 to 5 used to estimate the likelihood of a PET-CT finding being malignant, 5 being the most suspicious of malignancy [21]. In our sensitivity analysis, the Deauville score results were used for categorization of the cranial border and the external iliac region according to the NOAC guidelines and compared to the categorization based on the clinical staging. The sensitivity analysis was also used to determine whether the clinical assessment differed more from the non-biased retrospective evaluation in any of the predefined subgroups.

## Results

The mean age at diagnosis was 64.7 years and 80.1% were women. All patients were staged with CT, 91.6% with MRI and 97.0% with PET-CT (Table 2).

**Table 2:** Patient and tumor characteristics.

	*n* (%)
**Age at diagnosis, mean**	64.7
<70 years	120 (72.3)
≥70 years	46 (27.7)
**Female gender**	133 (80.1)
**Staging**	
CT	166 (100)
MRI	152 (91.6)
PET	161 (97.0)
**T stage**	
1	14 (8.4)
2	81 (48.8)
3	34 (20.5)
4	37 (22.3)
***N* stage**	
0	82 (49.4)
1a	64 (38.6)
1b	1 (0.6)
1c	19 (11.4)
**Site of lymph node metastasis**	
Perirectal^1^	28 (16.9)
Inguinal	62 (37.3)
Internal iliac	19 (11.4)
External iliac	20 (12.0)

1 Includes the mesorectal, presacral and superior rectal regions.

### Primary tumor

The primary tumor extended less than 1 cm into the rectum in 36 patients (21.7%) and more than 1 cm into the rectum in 68 patients (41.0%). In 62 patients (37.3%), no tumor growth above the anorectal junction was seen. Of the 131 patients having the primary tumor growth in relation to IRF assessed on MRI, 31 (23.7%) extended into the IRF, 44 (33.6%) extended into, but not beyond, the levator ani muscles or external sphincter and 56 (42.7%) did not extend into the levator ani muscles or external sphincter.

### Lymph node metastases

About half of the patients (50.6%) had LN metastasis with the inguinal region being the most common site (Table 2). There were 18 patients (10.8%) with LN metastasis in ≥3 and 66 patients (39.8%) with LN metastasis in <3 pelvic or inguinal LN regions. In total, 32 patients (19.3%) had LN metastasis in the internal or external iliac region, of which nine patients (5.4%) had LN metastasis in the upper half of the pelvis. Only three of these nine patients had LN metastasis in <3 pelvic or inguinal LN regions.

### Risk-adapted NOAC guidelines

Based on the assessment of the primary tumor and LN metastasis, 19.3% of the patients were categorized into the ‘Very low’, 8.4% into the ‘Low’, and 12.7% into the ‘High’ cranial border group, respectively. The remaining 59.6% were categorized as ‘Intermediate’ (Table 3).

**Table 3:** Contouring of the CTVe according to risk-adapted NOAC guidelines (n = 166).

	*n* (%)
**Cranial border**	
Very low	32 (19.3)
Low	14 (8.4)
Intermediate	99 (59.6)
High	21 (12.7)
**Inclusion of the external iliac region**	
Yes	98 (59.0)
No	68 (41.0)
**Inclusion of the ischiorectal fossa^1^**	
The entire ischiorectal fossa	31 (23.7)
2 cm margin into the ischiorectal fossa	44 (33.6)
No extra margin beyond the CTVp	56 (42.7)

1 Assessed for 131 patients with MRI available.

**Figure 3:**
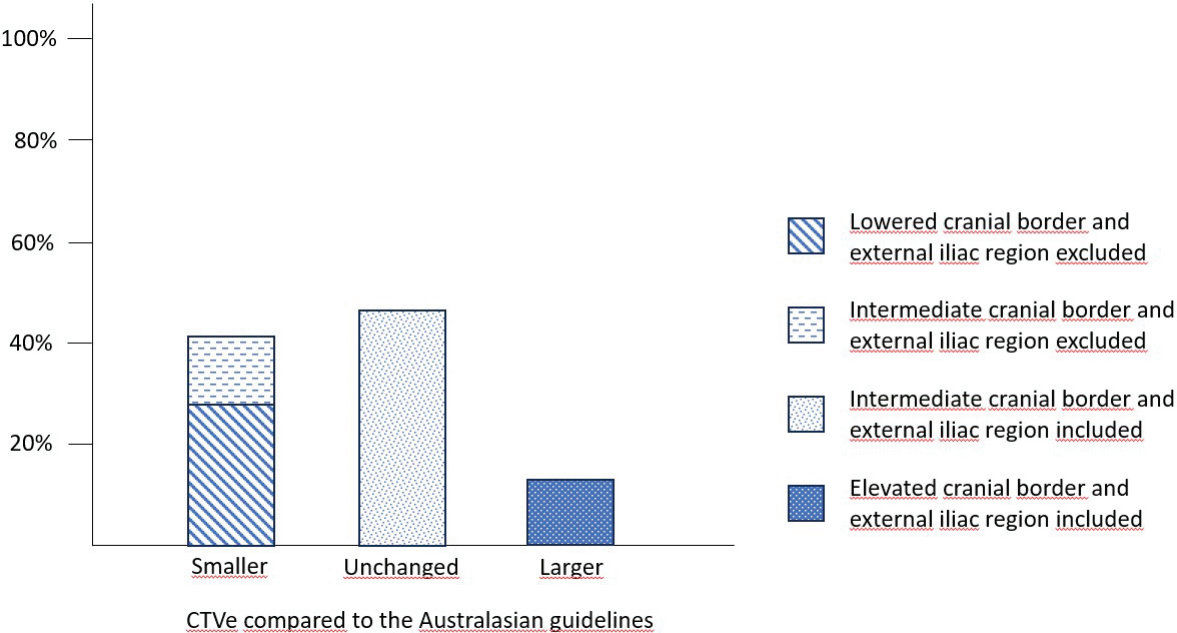
How the CTVe would change compared to the Australasian guidelines when the NOAC guidelines are applied.

The external iliac region was omitted in 68 patients (41.0%); 34 of these patients (20.5%) were staged T1-2N0 < 4 cm, 26 (15.7%) were staged T1-2N0 ≥ 4 cm, and 8 (4.8%) were staged T1-2N1 (perirectal LN metastases only). Combining information on the cranial border with information on the external iliac region, 41.0% of the patients had a smaller CTVe, 12.7% had a larger CTVe, and 46.3% had the same size of CTVe compared to the Australasian guidelines (Figure 3).

Based on the extension of the primary tumor, irradiation of the IRF could be omitted in 33.6% and partially reduced in 42.7% of patients, compared to the Australasian guidelines where inclusion of the entire IRF is recommended in all cases.

Defining pathological LNs with Deauville score instead of clinical staging had little impact on the cranial border and on the omission of the external iliac region (Supplementary Table S1).

### Gender and age

Statistical analysis showed no significant difference between men and women (Table 4). The external iliac region was omitted more often in patients aged ≥ 70 years than in patients aged < 70 years (60.9% vs. 33.3%; *p* = 0.001), which was driven by fewer T4 tumors (10.9 vs. 26.7%; *p* = 0.03) and more N0 patients (63.0 vs. 44.2%; *p* = 0.03) in the older age group. Using Deauville score (which should be unbiased to age) instead of clinical staging to define LN metastases, patients aged ≥ 70 years still had the external iliac region omitted more often than younger patients (56.8 vs. 35.7%; *p* = 0.015) (Supplementary Table S2).

**Table 4:** Age and gender differences.

	Age, *n* (%)			Gender, *n* (%)		
	<70 years	≥70 years	*P* ^2^	Female	Male	*P* ^2^
	120 (72.3)	46 (27.7)		133 (80.1)	33 (19.9)	
**Cranial border**			0.14			0.55
Very low *or* Low	29 (24.2)	17 (37.0)		38 (28.6)	8 (24.2)	
Intermediate	73 (60.8)	26 (56.5)		80 (60.2)	19 (57.6)	
High	18 (15.0)	3 (6.5)		15 (11.3)	6 (18.2)	
**Inclusion of the external iliac region**			0.001			0.16
Yes	80 (66.7)	18 (39.1)		75 (56.4)	23 (69.7)	
No	40 (33.3)	28 (60.9)		58 (43.6)	10 (30.3)	
**Inclusion of the ischiorectal fossa^1^**			0.11			0.98
Yes, the entire ischiorectal fossa	26 (28.3)	5 (12.8)		24 (23.3)	7 (25.0)	
2 cm margin into the ischiorectal fossa	31 (33.7)	13 (33.3)		35 (34.0)	9 (32.1)	
No extra margin beyond the CTVp	35 (38.0)	21 (53.8)		44 (42.7)	12 (42.9)	

1 Assessed for 131 patients with MRI available.

2 Using the chi-squared test.

## Discussion

This study was a retrospective simulation of how the CTVe would change in a cohort of anal cancer patients when applying the recently published risk-adapted NOAC contouring guidelines [18], instead of the previous Australasian standard. In 41.0% of the patients, the external iliac region was omitted and 27.7% had the cranial border lowered, whereas the cranial border was elevated in 12.7% when applying the NOAC guidelines.

Regarding the delineation of the IRF, there is currently no international consensus. The Australasian guidelines suggest inclusion of the entire IRF for all patients, and the UK guidelines recommend no extra margin beyond the primary tumor CTV [16, 22]. According to the NOAC guidelines, the entire IRF is only included if there is tumor extension beyond the levator ani muscles or the external sphincter, which 23.7% of patients in the present study had.

Another aim of our study was to investigate whether there were any age or gender groups that were more likely to have the cranial border changed or the external iliac region omitted. No significant gender differences were found, but patients with older age (≥70 years) were more likely to have the external iliac region omitted. Since the results are based on the clinical assessment at diagnosis, an objection could be that the clinicians, consciously or unconsciously, have tended to rule borderline LNs as benign rather than malignant in elderly patients. We therefore undertook a sensitivity analysis where LN status instead was based on an unbiased assessment using the PET derived Deauville score. Even though the strength of the association decreased slightly, the age difference was still significant in the sensitivity analysis.

The purpose of trying to reduce the CTVe is to decrease the radiation dose to OAR and, consequently, reduce toxicity. Previous anal cancer studies have suggested a correlation between acute gastrointestinal toxicity and radiation to the bowel cavity. The best dosimetric parameter for acute gastrointestinal toxicity seems to be bowel cavity V30Gy supported by Ng et al. [5], Devisetty et al. [10] and Nilsson et al. [9]. The later study by Nilsson et al. also suggests an association between late gastrointestinal toxicity and large bowel V20Gy. Lowering the cranial border of the CTVe as well as omission of the external iliac region are measures that will reduce the bowel volume exposed to radiation, and it seems like elderly patients more often get a decreased CTVe with the NOAC guidelines. Hopefully that can be beneficial since older patients are more prone to develop severe (grade ≥ 3) acute gastrointestinal toxicity [9, 23]. There are also other differences between the NOAC and the Australasian guidelines. For instance, the NOAC guidelines recommend only 5–7 mm around vessels and exclusion of bowel from part of the CTVe. Our next step will be to investigate the impact of all these changes combined on the radiation dose to OAR.

Our study is limited by its retrospective design and can only be seen as a description of how the NOAC guidelines are expected to be applied. Even though we have tried to be as precise as possible when defining the parameters within the radiological assessment that lay ground for the different risk groups, they are still assessments made by individuals with room for small variations of how the radiological findings are interpreted. There is no international consensus to define LN metastasis in anal cancer. In our study, we used the clinical assessments from the time at diagnosis, but we also undertook a sensitivity analysis with retrospective assessments using the Deauville score in a standardized way. The sensitivity analysis showed only small differences to the results based on the clinical assessment at diagnosis, suggesting that the results are relatively robust. In the NOAC guidelines, CTVe contouring is based on tumor stage and our results would accordingly be affected by the case mix in the study population. The cohort used in the present study was a consecutive series including all patients referred to a single center during 2009–2017. The distribution of age, gender and stage was very similar to a recent large Swedish registry study that also describes current treatment schemes and radiation doses in Sweden [4], indicating that our study population was a representative mix of patients with anal cancer in daily practice.

The results of this study suggest that using the NOAC risk-adapted contouring guidelines will change, and in most cases reduce, the CTVe in about half of all anal cancer patients treated curatively with CRT compared with previous guidelines. To what extent that will result in a clinically meaningful reduction of toxicity and if the volume reduction is safe in terms of oncological outcomes are yet to be investigated. Anal cancer patients in Sweden are treated according to the risk-adapted NOAC guidelines since September 2023, and prospective follow-up of these patients is important to answer these questions.

## Supplementary Material

Supplementary material has been published as submitted. It has not been copyedited, or typeset by Acta Oncologica

## Data Availability

The present data are summarized in this paper. The complete dataset can be retrieved from the corresponding author on reasonable request.
