# Supplementary material for: Impact of the risk-adapted Nordic anal cancer group consensus guidelines on the contouring of the elective clinical target volume in anal cancer
Source: Acta Oncol. 2025 May 26;64:42723. doi: 10.2340/1651-226X.2025.42723 (PMC12128630; doi:10.2340/1651-226X.2025.42723)
Supplement: Supplementary material has been published as submitted. It has not been copyedited, or typeset by Acta Oncologica [file AO-64-42723-s1.pdf]

Supplementary material has been published as submitted. It has not been copyedited, or typeset by Acta Oncologica

Table S1. Contouring of the CTVe according to risk-adapted NOAC guidelines

	Clinical staging[1] *n* (%)	Deauville score 4-5[2] *n* (%)	Deauville score 3-5[2] *n* (%)
**Cranial border of CTV**			
Very low	32 (19.3)	32 (20.1)	27 (17.0)
Low	14 (8.4)	15 (9.4)	15 (9.4)
Intermediate	99 (59.6)	93 (58.5)	93 (58.5)
High	21 (12.7)	19 (11.9)	24 (15.1)
**Inclusion of external iliac region**			
Yes	98 (59.0)	93 (58.5)	98 (61.6)
No	68 (41.0)	66 (41.5)	61 (38.4)

[1] All patients (*n* = 166)

[2] Assessed for 159 patients with PET-CT available

Table S2. Age differences between clinical staging and retrospective PET-CT assessment

	Clinical staging[1]			Deauville score 4-5[2]			Deauville Score 3-5[2]		
	Age, *n* (%)			Age, *n* (%)			Age, *n* (%)		
	< 70 years	≥ 70 years	*P*[3]	< 70 years	≥ 70 years	*P*[3]	< 70 years	≥ 70 years	*P*[3]
	120 (72.3)	46 (27.7)		115 (72.3)	44 (27.7)		115 (72.3)	44 (27.7)	
**Cranial border**			0.14			0.65			0.53
Very low *or* Low	29 (24.2)	17 (37.0)		32 (27.8)	15 (34.1)		28 (24.3)	14 (31.8)	
Intermediate	73 (60.8)	26 (56.5)		68 (59.1)	25 (56.8)		68 (59.1)	25 (56.8)	
High	18 (15.0)	3 (6.5)		15 (13.0)	4 (9.1)		19 (16.5)	5 (11.4)	
**Inclusion of the external iliac region**			0.001			0.015			0.009
Yes	80 (66.7)	18 (39.1)		74 (64.3)	19 (43.2)		78 (67.8)	20 (45.5)	
No	40 (33.3)	28 (60.9)		41 (35.7)	25 (56.8)		37 (32.2)	24 (54.5)	

[1] All patients (*n* = 166)

[2] Assessed for 159 patients with PET-CT available

[3] Using the chi-squared test
